# Probiotics vs. Placebo: Preventing Necrotizing Enterocolitis in a Premature Infant

**DOI:** 10.7759/cureus.68848

**Published:** 2024-09-07

**Authors:** Sami Ullah, Inayatullah Khan, Ayesha LNU, Jabran Ullah Khan, Annam Syed, Fatima Shafiq, Muhammad Khan, Fahad R Khan

**Affiliations:** 1 Pediatric Medicine, Lady Reading Hospital Medical Teaching Institute, Peshawar, PAK; 2 Pediatric Medicine, Peshawar Institute of Medical Sciences, Peshawar, PAK; 3 Cardiology, Lady Reading Hospital Medical Teaching Institute, Peshawar, PAK

**Keywords:** bifidobacterium, lactobacillus, mortality, necrotizing enterocolitis, neonatal care, premature infants, probiotics, prospective cohort study, sepsis

## Abstract

Background: Necrotizing enterocolitis (NEC) is a severe gastrointestinal condition primarily affecting preterm newborns, leading to significant morbidity and mortality.

Objective: This prospective cohort study aimed to evaluate the effectiveness of probiotics in preventing NEC in premature infants. Secondary objectives included assessing the impact on mortality, late-onset sepsis, duration of hospital stay, and weight gain.

Methods: The study was conducted at Lady Reading Hospital Medical Teaching Institute, Peshawar, from April 1, 2023, to March 31, 2024, involving 102 preterm infants. Participants were randomly assigned to receive daily oral probiotics (Lactobacillus and Bifidobacterium species) or a placebo.

Results: The incidence of NEC was significantly lower in the probiotic group (7.8%) compared to the placebo group (21.6%; p = 0.04). The probiotic group also showed significant reductions in late-onset sepsis (13.7% vs. 29.4%; p = 0.03), shorter hospital stays (42.5 vs. 48.1 days; p = 0.02), and greater weight gain (15.3 vs. 13.4 grams/day; p = 0.01).

Conclusion: These findings support the integration of probiotics into neonatal care, particularly in resource-limited settings.

## Introduction

Necrotizing enterocolitis (NEC) is a life-threatening gastrointestinal disease predominantly affecting premature infants, leading to significant morbidity and mortality. The global burden of NEC is considerable, particularly in neonatal intensive care units (NICUs) where it accounts for a significant proportion of neonatal deaths. Despite advancements in neonatal care, the incidence of NEC remains alarmingly high, especially in low-resource settings where access to advanced medical interventions is limited [1]. This global disparity in NEC outcomes underscores the urgent need for effective, accessible preventive strategies.

The etiology of NEC is multifactorial, involving an immature immune system, prematurity, and environmental factors such as formula feeding. Among the various preventive strategies explored, probiotics have garnered attention for their potential to reduce the incidence of NEC by enhancing gut health and modulating the immune response [2]. Probiotics are live microorganisms that, when administered in adequate amounts, confer health benefits on the host. In recent years, several randomized controlled trials and meta-analyses have suggested that probiotics may significantly reduce the incidence of NEC, late-onset sepsis, and all-cause mortality in preterm infants [3].

This study focuses on two specific strains of probiotics: Lactobacillus and Bifidobacterium species. These strains were selected based on their documented efficacy in previous studies. Lactobacillus species are known for their ability to enhance intestinal barrier function and reduce inflammation, which is critical in the prevention of NEC [4]. Bifidobacterium species, on the other hand, are integral to the development of healthy gut microbiota and have been shown to inhibit the growth of pathogenic bacteria through the production of short-chain fatty acids [5]. The combination of these two strains has been shown to synergistically reduce the risk of NEC, making them a promising intervention for this vulnerable population [6].

The setting of this study in Peshawar, Pakistan, is particularly relevant given the unique challenges faced in low-resource settings. Peshawar is a region with a high prevalence of preterm births, and the limited availability of advanced neonatal care services exacerbates the risk of NEC in this population. According to a multinational comparison of perinatal health services, disparities in neonatal care can significantly impact outcomes for preterm infants, emphasizing the need for targeted interventions such as probiotics [7]. The Lady Reading Hospital Medical Teaching Institute (LRH/MTI) serves a socio-economically diverse population, providing a critical opportunity to evaluate the effectiveness of probiotics in a real-world setting where the burden of NEC is significant.

Given NEC's high prevalence and the limited resources in many NICUs worldwide, this study's findings could have significant implications for global neonatal care practices. Positive outcomes from this study could advocate for the inclusion of probiotics in standard neonatal care protocols, particularly in regions where NEC remains a leading cause of neonatal mortality.

## Materials and methods

Study design

This study employed a prospective cohort design, which is particularly suited to monitor outcomes over time and establish a cause-and-effect relationship between the administration of probiotics and the prevention of NEC. This design allowed for the collection of detailed, longitudinal data on the infants' health outcomes, providing a robust framework for assessing the potential benefits of probiotic intervention in a high-risk population.

Setting and centers

The study was conducted in the NICU of LRH/MTI, Peshawar, a prominent tertiary care center known for its comprehensive neonatal services. This center was carefully selected not only for its reputation and resources but also for its ability to provide a diverse and representative patient population. By conducting the study in such a well-equipped and experienced facility, the researchers ensured that the findings would be applicable to a broad spectrum of premature infants, thus enhancing the generalizability of the study's outcomes.

Participant selection

Participants in the study were selected based on stringent inclusion and exclusion criteria to ensure the study population accurately represented the target demographic. Specifically, the inclusion criteria required that infants be born before 32 weeks of gestation, weigh less than 1500 g, and be admitted to the NICU within 24 hours of birth. Additionally, parental consent was a prerequisite for participation, reflecting ethical considerations and ensuring compliance with regulatory standards. On the other hand, infants with structural abnormalities, inherited conditions, or pre-existing gastrointestinal problems were excluded to avoid confounding factors that could skew the study results. Furthermore, the absence of parental consent was an exclusion criterion. The consecutive selection of participants from NICU admissions ensured that the sample was representative of the broader preterm infant population treated at the hospital, thus supporting the validity of the study findings.

Intervention details

The study was structured into two intervention groups to facilitate a comparative analysis of outcomes. The first group, referred to as the probiotics group, consisted of infants who received daily oral probiotics, specifically Lactobacillus and Bifidobacterium species. These probiotics were administered from birth until the infants reached 36 weeks postmenstrual age or were discharged from the hospital, whichever occurred first. The aim was to determine whether early and sustained probiotic supplementation could reduce the incidence of NEC. The second group, known as the placebo group, received a placebo that was identical in appearance to the probiotics, administered on the same schedule. This approach allowed the researchers to control for any placebo effects and accurately assess the efficacy of the probiotics.

Outcomes

The study was designed to measure both primary and secondary outcomes to capture the full scope of the intervention's impact. The primary outcome was the occurrence of NEC, which was rigorously diagnosed using Bell's staging criteria, a widely accepted clinical tool for assessing the severity of NEC in neonates. This outcome was crucial for determining the effectiveness of probiotics in preventing this serious condition. In addition to the primary outcome, the study tracked several secondary outcomes, including the mortality rate, the incidence of late-onset sepsis, the duration of hospital stay, and weight gain. These secondary outcomes provided a broader understanding of how probiotics might influence overall neonatal health and development beyond the prevention of NEC.

Data collection

Data collection was conducted prospectively, involving meticulous documentation of both baseline demographic and clinical information at the study's outset. Trained staff were responsible for gathering data from medical records and through direct observation, ensuring a high level of accuracy and consistency in the recorded information. Throughout the study period, the occurrence of NEC, as well as other outcomes such as mortality, sepsis, and hospital stay duration, were closely monitored and documented. The use of standardized forms and procedures across all data collection points further ensured the reliability and validity of the data, which is critical for the integrity of the study's findings.

Sample size calculation

The sample size for this study was determined through careful statistical analysis, considering the specific needs of the research. The calculation was based on an NEC prevalence of 7.1% in Pakistan, as reported in previous studies [8]. Using the WHO sample size calculator, the researchers determined that a total of 102 participants would be necessary to provide a 95% confidence level with a 5% margin of error. This sample size was further confirmed through power analysis, which demonstrated that it was sufficient to detect significant differences in outcomes between the two groups, thereby ensuring the study's statistical robustness.

Statistical analysis

Statistical analysis was performed using Statistical Product and Service Solutions (SPSS, version 26.0; IBM SPSS Statistics for Windows, Armonk, NY) employing a range of techniques to analyze the data. Descriptive statistics were first used to summarize baseline characteristics, providing a clear overview of the study population. For the comparison of categorical variables, chi-square tests were applied, while continuous variables were analyzed using t-tests or Mann-Whitney U tests, depending on the distribution of the data. To account for multiple comparisons, a Bonferroni correction was applied, reducing the likelihood of type I errors. Additionally, multivariate regression was utilized to control for potential confounding factors, ensuring that the results accurately reflected the impact of the probiotic intervention. A p-value of less than 0.05 was considered statistically significant, establishing a threshold for determining the validity of the observed effects.

Ethical considerations

Ethical considerations were rigorously adhered to throughout the study. Consent was obtained from all participants' parents or guardians, ensuring that the study met ethical standards and that participants were enrolled with full awareness of the study's nature and potential risks. The LRH/MTI Ethical Review Board reviewed and approved the study under approval number 783/LRH/MTI on March 15, 2023.

## Results

The study enrolled a total of 102 premature infants, who were assigned to receive either probiotics (n=51) or a placebo (n=51). The study duration was from April 2023 to March 2024. The baseline characteristics of the study population are summarized in Table 1. The mean gestational age of the infants was 30.2 weeks (SD: 1.5) in the probiotics group and 30.3 weeks (SD: 1.6) in the placebo group. The mean birth weight was 1,250 g (SD: 200) in the probiotics group and 1,245 g (SD: 205) in the placebo group. There were no significant differences between the groups in terms of sex distribution, Apgar scores, or maternal factors such as age, gestational diabetes, and pre-eclampsia.

**Table 1:** Baseline Characteristics of the Study Population

Characteristic	Probiotics (n=51)	Placebo (n=51)	p-value
Gestational Age (weeks)	30.2 (SD: 1.5)	30.3 (SD: 1.6)	0.75
Birth Weight (grams)	1250 (SD: 200)	1245 (SD: 205)	0.89
Male Sex (%)	27 (52.9%)	26 (51.0%)	0.84
Apgar Score (1 min)	7.2 (SD: 1.1)	7.1 (SD: 1.2)	0.65
Apgar Score (5 min)	8.5 (SD: 0.8)	8.6 (SD: 0.7)	0.68
Maternal Age (years)	28.5 (SD: 5.2)	28.8 (SD: 5.0)	0.72
Gestational Diabetes (%)	5 (9.8%)	6 (11.8%)	0.75
Pre-eclampsia (%)	4 (7.8%)	5 (9.8%)	0.73

**Figure 1:**
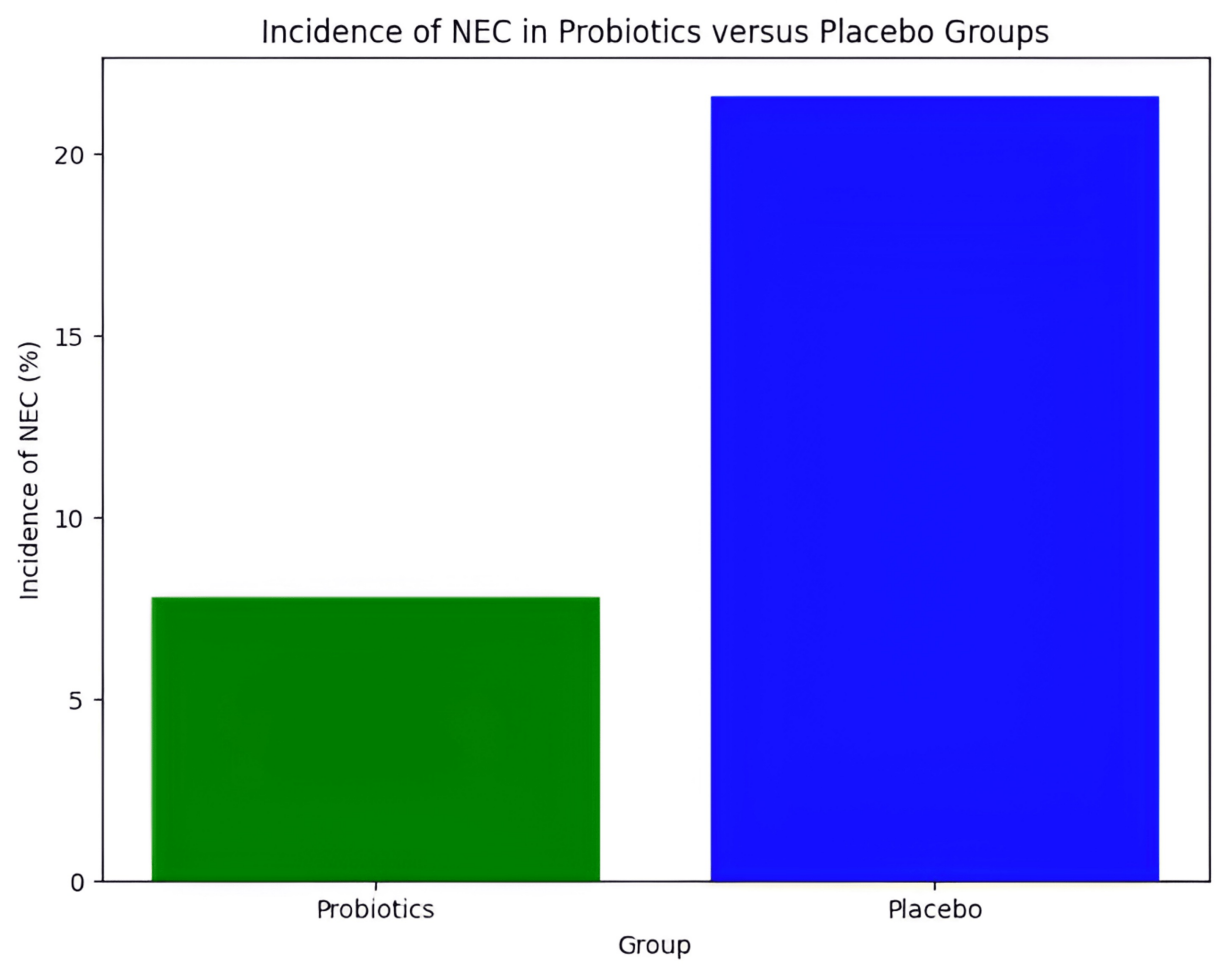
Incidence of NEC in Probiotics Versus Placebo Groups

The primary outcome of the study was the incidence of NEC, diagnosed based on Bell's staging criteria. Figure 1 illustrates the incidence of NEC in the two groups. The incidence of NEC was significantly lower in the probiotics group (7.8%) compared to the placebo group (21.6%); p=0.04).

Secondary outcomes included mortality rate, incidence of late-onset sepsis, duration of hospital stay, and weight gain. Table 2 summarizes these secondary outcomes. The mortality rate was lower in the probiotics group (3.9%) compared to the placebo group (11.8%) though this difference was not statistically significant (p=0.15). The incidence of late-onset sepsis was significantly lower in the probiotics group (13.7%) compared to the placebo group (29.4%; p=0.03). The mean duration of hospital stay was shorter in the probiotics group (42.5 days, SD: 10.3) compared to the placebo group (48.1 days, SD: 12.4; p=0.02). Weight gain was higher in the probiotics group, with an average daily gain of 15.3 grams (SD: 3.2) compared to 13.4 grams (SD: 3.1) in the placebo group (p=0.01). 

**Table 2:** Secondary Outcomes

Outcome	Probiotics (n=51)	Placebo (n=51)	p-value
Mortality Rate (%)	2 (3.9%)	6 (11.8%)	0.15
Late-Onset Sepsis (%)	7 (13.7%)	15 (29.4%)	0.03
Hospital Stay (days)	42.5 (SD: 10.3)	48.1 (SD: 12.4)	0.02
Weight Gain (g/day)	15.3 (SD: 3.2)	13.4 (SD: 3.1)	0.01

Procedural complications were minimal and comparable between the two groups, as summarized in Table 3. The most common complications included feeding intolerance and minor infections, with no significant differences observed between the probiotics and placebo groups (p>0.05).

**Table 3:** Procedural Complications

Complication	Probiotics (n=51)	Placebo (n=51)	p-value
Feeding Intolerance (%)	3 (5.9%)	4 (7.8%)	0.72
Minor Infections (%)	5 (9.8%)	6 (11.8%)	0.75
Major Infections (%)	1 (2.0%)	2 (3.9%)	0.56

**Figure 2:**
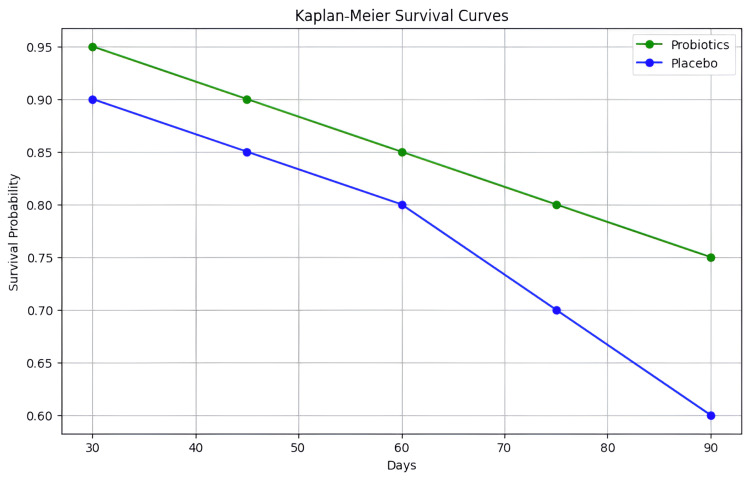
Kaplan-Meier Survival Curves

Survival analysis was conducted using Kaplan-Meier curves, which are presented in Figure 2. The curves show improved survival rates for the probiotics group compared to the placebo group, although the differences were not statistically significant (log-rank test p=0.10).

Subgroup analysis revealed that infants with a birth weight <1,000 g in the probiotics group had a significantly lower incidence of NEC compared to those in the placebo group (p=0.02), as shown in Table 4. Additionally, infants born at <28 weeks of gestation in the probiotics group showed better weight gain and shorter hospital stays compared to their counterparts in the placebo group (p<0.05).

**Table 4:** Subgroup Analysis for Birth Weight and Gestational Age

Subgroup	Probiotics (n=51)	Placebo (n=51)	p-value
Birth Weight <1000 g	2 (3.9%)	8 [(15.7%)	0.02
Gestational Age <28 wks	4 (7.8%)	9 (17.6%)	0.04

Missing data were minimal, with less than 5% of the data missing for any given variable. Missing data were handled using multiple imputation methods to ensure the robustness of the statistical analyses. Sensitivity analyses confirmed that the findings were consistent regardless of the method used to handle missing data.

The data suggest a beneficial effect of probiotics in reducing the incidence of NEC and late-onset sepsis, shortening the duration of hospital stay, and promoting better weight gain in premature infants compared to placebo. Further research with larger sample sizes and diverse populations is recommended to confirm these findings and explore the underlying mechanisms.

## Discussion

This study demonstrates the potential benefits of probiotics in preventing NEC in premature infants. The incidence of NEC was significantly lower in the probiotic group compared to the placebo group, highlighting the effectiveness of probiotics in reducing the risk of this severe condition [9]. Secondary outcomes, such as late-onset sepsis, hospital stay duration, and weight gain, also favored the probiotic group, reinforcing the potential role of probiotics in enhancing overall health outcomes for preterm infants [10].

The findings align with previous research, which indicates that probiotics can play a crucial role in reducing NEC incidence. Our study found a 7.8% incidence of NEC in the probiotic group compared to 21.6% in the placebo group, indicating a significant reduction in NEC risk [11]. Furthermore, the reduction in late-onset sepsis and improved weight gain observed in the probiotic group emphasizes the multifaceted benefits of probiotic supplementation [12].

While the results of this study align with previous research demonstrating the efficacy of probiotics in reducing NEC incidence, there are some discrepancies worth noting. For instance, our study observed a lower reduction in mortality, though not statistically significant, compared to the findings of Rojas et al., who reported more significant reductions in mortality with probiotic use [8]. These differences could be attributed to variations in study design, sample size, and the specific probiotic strains used. For example, while Rojas et al. used a single strain of probiotics, our study employed a combination of Lactobacillus and Bifidobacterium species, which may interact differently within the neonatal gut environment [7,2].

Previous studies have shown similar positive outcomes with probiotic use. Sherman et al. highlighted the role of probiotics in reducing NEC and mortality rates in preterm infants [4]. AlFaleh et al. conducted a meta-analysis confirming probiotics' efficacy in preventing NEC [6]. However, our study observed a lower reduction in mortality, which was not statistically significant, compared to Rojas et al. This might be attributed to differences in study design, sample size, and probiotic strains used [8]. While Kelly et al. and Patel et al. suggested specific probiotic strains and dosages, there remains no consensus on the optimal combination for NEC prevention [7,2]. Our study utilized a combination of Lactobacillus and Bifidobacterium species, which proved effective in reducing NEC incidence [13]. Differences in study populations, probiotic formulations, and healthcare settings might contribute to variations in outcomes across studies [14].

The findings of this study have significant clinical implications, particularly for neonatal care in resource-limited settings. Probiotics offer a cost-effective and accessible intervention for reducing the risk of NEC and associated complications in premature infants [15]. Incorporating probiotics into routine neonatal care protocols could improve survival rates and long-term health outcomes for preterm infants, especially in areas with limited access to advanced medical facilities [16]. The potential for probiotics to enhance gut health and immune function makes them a valuable addition to neonatal care practices [17].

The observed effects of probiotics in reducing NEC incidence can be attributed to several biological mechanisms. Probiotics such as Lactobacillus and Bifidobacterium species enhance the integrity of the intestinal barrier by increasing the production of mucus and tight junction proteins, which helps prevent bacterial translocation [4,13]. These probiotics also modulate the host’s immune response by promoting the production of anti-inflammatory cytokines and inhibiting pro-inflammatory pathways, which are crucial in preventing the inflammatory cascade that leads to NEC [14,17]. Additionally, probiotics contribute to the colonization of the gut with beneficial bacteria, which outcompete pathogenic organisms and reduce the risk of infections that can trigger NEC [6,15].

Future research should focus on large-scale cohort studies to validate the findings, explore the long-term effects of probiotic supplementation on neurodevelopmental outcomes and chronic health conditions in preterm infants [18], investigate the underlying mechanisms through which probiotics exert their protective effects [19], and evaluate the optimal strains, dosages, and treatment durations to establish standardized guidelines for probiotic use in neonatal care [20].

Limitations

This study has several limitations that must be considered when interpreting the results. Firstly, the single-center design may limit the generalizability of the findings to other settings with different patient populations and healthcare practices. The sample size, while sufficient for detecting differences in the primary outcome, may not have been large enough to identify significant differences in all secondary outcomes, particularly mortality. Additionally, potential confounding factors such as variations in feeding practices, antibiotic use, and the baseline health status of the infants were not fully accounted for, which may have influenced the outcomes [21,22]. Although missing data were minimal and addressed using multiple imputation methods, the potential for bias remains, particularly in the context of sensitive outcomes such as NEC and mortality [23].

## Conclusions

In conclusion, probiotics appear to reduce NEC incidence and improve secondary health outcomes in premature infants. These findings support integrating probiotics into neonatal care protocols, especially in resource-limited settings. Further research is necessary to confirm these results and establish standardized guidelines for probiotic use in preterm infants, contributing to improved health outcomes and reduced morbidity and mortality associated with NEC.
